# A tough act to follow: collagen hydrogel modifications to improve mechanical and growth factor loading capabilities

**DOI:** 10.1016/j.mtbio.2021.100098

**Published:** 2021-02-12

**Authors:** S.O. Sarrigiannidis, J.M. Rey, O. Dobre, C. González-García, M.J. Dalby, M. Salmeron-Sanchez

**Affiliations:** Centre for the Cellular Microenvironment, University of Glasgow, Glasgow G12 8LT, UK

**Keywords:** Extracellular matrix, Hydrogels, Growth factor delivery, Mechanical properties, Tissue engineering

## Abstract

Collagen hydrogels are among ​the most well-studied platforms for drug delivery and *in situ* tissue engineering, thanks to their low cost, low immunogenicity, versatility, biocompatibility, and similarity to the natural extracellular matrix (ECM). Despite collagen being largely responsible for the tensile properties of native connective tissues, collagen hydrogels have relatively low mechanical properties in the absence of covalent cross-linking. This is particularly problematic when attempting to regenerate stiffer and stronger native tissues such as bone. Furthermore, in contrast to hydrogels based on ECM proteins such as fibronectin, collagen hydrogels do not have any growth factor (GF)-specific binding sites and often cannot sequester physiological (small) amounts of the protein. GF binding and *in situ* presentation are properties that can aid significantly in the tissue regeneration process by dictating cell fate without causing adverse effects such as malignant tumorigenic tissue growth. To alleviate these issues, researchers have developed several strategies to increase the mechanical properties of collagen hydrogels using physical or chemical modifications. This can expand the applicability of collagen hydrogels to tissues subject to a continuous load. GF delivery has also been explored, mathematically and experimentally, through the development of direct loading, chemical cross-linking, electrostatic interaction, and other carrier systems. This comprehensive article explores the ways in which these parameters, mechanical properties and GF delivery, have been optimized in collagen hydrogel systems ​and examines their *in vitro* or *in vivo* biological effect. This article can, therefore, be a useful tool to streamline future studies in the field, by pointing researchers into the appropriate direction according to their collagen hydrogel design requirements.

## Graphical abstract

**Figure:**
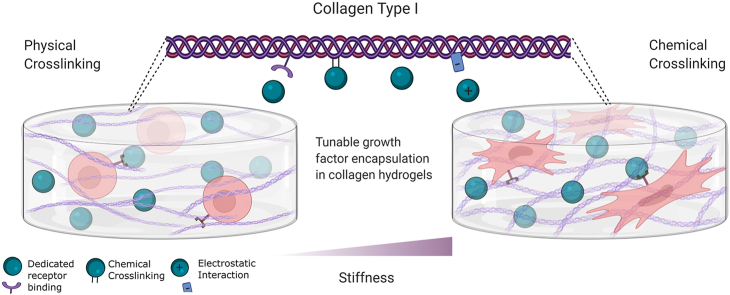

## 1 Introduction

**Fig. 1:**
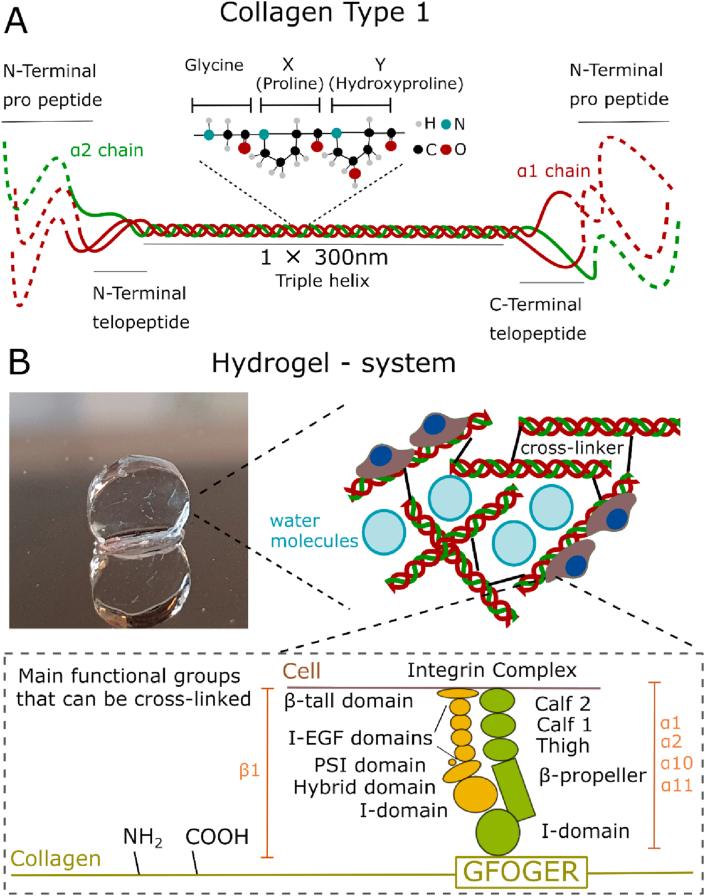
**A Type 1 collagen structure** ​shows the triple helix (made up of two α1 and one α2 chains) and the N- and C-terminal telo- and pro-peptides of a collagen type I molecule. B Collagen type I is gelled using various methods discussed later to form a hydrogel system. Cross-linking is carried out by taking advantage of collagen's free amine and carboxyl groups. Example of collagen–cell interaction through GFOGER-integrin ligation.

Collagen is the most abundant protein in the animal kingdom and is a key component of the extracellular matrix (ECM). There are at least 28 members that belong to the collagen superfamily; however, the defining characteristics of collagen can be quite loose. Over 90% of collagen in the human body is either type I, II, or III [1]. This article will focus on collagen type I, the most widely used collagen in tissue engineering, which is made up of three alpha chains (two α1 and one α2). Some exist in the form of an α1 homotrimer albeit in small amounts [1]. Each collagen chain is made up of approximately 1000 amino acids following a Gly-X-Y repeating sequence. X and Y being usually proline and hydroxyproline, respectively [2] (Fig. 1).

Because of its ubiquity, collagen is also easily and cheaply isolated from tissues such as skin, tendon, pericardium, and other and is widely used as a biomaterial, such as hydrogels (materials with a water content >90%), for tissue regeneration. Commonly it is extracted not only from bovine, porcine, or rat specimens ​but also from marine or recombinant sources [3]. It can be isolated using acid or enzymes such as pepsin and can be found in two forms: as atelocollagen (without telopeptides) or telocollagen (with telopeptides) (Fig. 1). The free functional groups of collagens (amines and carboxyl) can be used to modify their structure and be used to create physical or chemical cross-links [4] (Fig. 1). The ability of collagen to form intra- and interfibrillar cross-links can be exploited to create hydrogels with a range of mechanical properties to match the ones showcased by the surrounding tissue when implanted. A general compilation of cross-linking strategies for hydrogels and other biomaterials has been undertaken by other authors [5,6]. However, the impact on the mechanical properties of collagen hydrogels has only been reviewed considering mainly non-covalent cross-linking mechanisms [7,8], or specialized applications such as 3D printing [9,10].

Collagen is also a highly biocompatible material, which provides the ideal environment for cell attachment and proliferation [11], making it a great candidate for use in tissue regeneration. It is mostly broken down through enzymatic pathways, in contrast to synthetic polymers, which degrade hydrolytically into cytotoxic by-products. Cells bind to the GFOGER domain through integrins α1β1, α2β1, α10β1, and α11β1 [11] (Fig. 1). To increase the bioactivity of collagen hydrogels, researchers have used them in conjunction with growth factors (GFs) e.g. BMP-2-soaked collagen hydrogel scaffolds for bone regeneration. GFs are proteins involved in many cellular processes and intercellular communication. They can induce cell proliferation, maturation, and differentiation [12]. However, collagen has limited GF binding properties compared with other ECM proteins such as fibronectin or laminin [13]. Thus, research has focused on various novel methods to overcome this obstacle.

In this article, we will overview the various techniques used to produce collagen hydrogels with varying mechanical and degradation properties for different tissue engineering applications. We will explore the different collagen-based, hydrogel GF delivery systems that researchers have developed in recent years and the successful *in vitro* and *in vivo* experiments that have been achieved with collagen hydrogels. GF delivery has only recently been explored empirically and through mathematical modeling, particularly in conjunction with mechanical properties, and has immense potential use in biomaterials in tissue regeneration. Mechanical property optimization and GF delivery are of major importance in achieving tissue regeneration, thus it is important to review them in detail. Extensive reports on collagen hydrogel systems with and without GFs cannot be found in literature yet. Given that collagen is an important biomaterial used extensively in tissue engineering, this article is important in guiding future research in the field.

## 2 Mechanical considerations of collagen hydrogels

Biomaterial selection for a particular application often requires mechanical properties of constructs similar to the tissue where they are implanted, as stiffness is known to determine cell behavior through mechanosensitive cell receptors. Despite their advantages, collagen's rapid degradation rate (hence weak mechanical strength), opacity or high shrinkage have limited its extended application for tissue engineering [14,15] also affecting clinical usability in tissues, which may require higher stiffness, like bone or cartilage. To give an example post-treatment or cross-linking of collagen hydrogels can increase the stiffness of the matrices, stimulating mesenchymal stem cell (MSC) differentiation into osteogenic lineages, which has been proposed to occur at Young's modulus above 25 ​kPa from atomic force microscopy (AFM) measurements [16]. The formation of additional cross-links in hydrogels prevents collagen molecules from sliding past each other under stress [17]. This increases the mechanical properties of the cross-linked hydrogels, in terms of tensile, compressive, and shear elastic moduli [17]. Cross-link density, apart from the strength of the cross-links or presence of multifunctional groups, is thought to be a major contributor to collagen matrix stiffness [18,19]. Modifying the mechanical properties of a hydrogel in a single direction, thus creating anisotropic hydrogels, can also be critical to foster tissue growth in a preferred direction in applications like neural tissue regeneration or angiogenesis [20,21]. The combination of natural-based hydrogels with adjuncts that can enhance their mechanical properties, such as nanoparticle loading (which includes graphene nanoparticles, Au, dendrimers), or combining with synthetic fibers or 3D scaffolds (PCL, PGA, etc.) are another fundamental approach for increasing the mechanical properties of soft hydrogels. The reader can consult the comprehensive reviews by Thoniyot et al. and Tozzi et al. [22,23]*,* and more recently Fathi-Achachelouei et al. [24], where several examples of collagen hydrogels and their combination with nanoparticles or with solid scaffolds are included. It is also important to consider hydrogel degradation in the presence of metalloproteinases including collagenase, that are found in native tissues, as it can also give valuable information about the expected mechanical stiffness once the hydrogels are implanted. The usual concentration of collagenase used *in vitro* lies between 0.1 and 5 ​U/ml, although this may depend on the final intended application of the hydrogels [[25], [26], [27], [28]]. The modulation of collagen hydrogels' mechanical properties can therefore expand their applicability to a wider range of *in vitro* and *in vivo* applications (summarized in Table 1).

**Table 1:** Collagen hydrogel cross-linking systems and their resulting properties: summary of the main parameters examined, which resulted in varying mechanical properties of the hydrogels and their *in vivo/in vitro* behavior.

Mechanism of cross-linking	Parameters varied	Network properties SEM: Scanning electron microscopy TEM: Transmission emission microscopy CRM: Confocal reflectance microscopy MPM: Multiphoton microscopy	Mechanical properties	*In vitro/In vivo* studies	References
**Physical cross-linking**					
Fibrillogenesis	Collagen concentration	N/A	Shear moduli	Cytotoxicity, enhanced endothelial/osteogenic differentiation, morphology of dental progenitor stem cells by combining soft and stiffer hydrogel.	[78]
		N/A	Shear moduli	Dorsal root ganglia seeding, increased neurite length and number, at low stiffness and collagen concentration	[77]
	Collagen concentration, source (bovine, porcine, rat)	Fibril width and volume fraction (CRM)	Gelification rate, turbidity, shear, compression and tensile moduli	MSC differentiation, adipocyte proliferation at a stiffness of 45 ​Pa or osteogenesis (calcium nodules) at 700 ​Pa	[76]
	pH	Fibril width (SEM)	Relaxation moduli, compression test	Focal adhesions (actin, vinculin), wider endothelial cell network formation in rigid gels	[66]
	Temperature	Fibril width (MPM, SEM)	Shear moduli (rheology)	No	[65]
		Fibril width, pore size (CRM, SEM)	Shear moduli (rheology)	Greater stiffness/larger pore size promotes cell contractility, local matrix remodeling, and differentiation into proangiogenic myofibroblasts. Lumen formation was only observed in cold cast hydrogels mixed with Matrigel, also increased vessel branching	[83] [82]
	Collagen concentration, pH, polymerization time	Fibril width (CRM)	Tensile stress (linear moduli, failure stress/strain)	No	[68]
	Collagen and salts concentration, pH, temperature	Fibril width (SEM), zeta potential	Gelification rate, turbidity	No	[51,61]
	Collagen concentration, pH, temperature	Fibril width, pore size (CRM), diffusivity	Gelification rate, turbidity, compression moduli (confined)	No	[63]
		Fibril width (SEM)	Turbidity, tensile moduli, ultimate tensile stress, compressive moduli	Cytotoxicity of porcine smooth muscle cells	[64]
	Fiber alignment	Fibril width (SEM, CRM), orientation (light microscope)	Gelification rate, turbidity, shear moduli	Increased neuronal growth and neurite elongation in aligned gels	[85]
		Fibril width (bright field)	Tensile moduli	Myoblasts aligned with collagen fibrils	[95]
		Fibril orientation (CRM), optical transmittance	Viscosity	Different keratonocyte fate depending on degree of alignment *in vitro*. Similar fibril structure to native cornea after 4 weeks *in vivo*	[97]
	Fiber alignment, collagen concentration	Fibril orientation (CRM)	Shear moduli	LIVE/DEAD, neurite growth and orientation, electrophysiological activity	[89]
				Increased myotube formation in low stiffness matrices; accelerated muscle function recovery in rat laryngectomy mode	[92]
UV cross-linking	UV cross-linking time	Contraction rate	Tensile strength	Keratinocyte proliferation similar to GA cross-linking	[109]
	UV irradiation dose	Macromolecule size (chromatography)	Shear moduli	No	[111]
	UV cross-linking time, riboflavin concentration	Contraction rate, triple helix structure (circular dichroism analysis)	Shear moduli, swelling, degradation	Enhanced gene expression levels for the collagen II and aggrecan with encapsulated chondrocytes	[112]
**Chemical cross-linking**					
Glutaraldehyde	Cross-linker concentration	Fibril size (TEM)	Compressive moduli, denaturation temperature	No	[135]
Isocyanates	Cross-linker concentration, cross-linking time	Cross-linking degree	Tensile moduli, elongation at break	No	[136]
	N/A	N/A	Shear moduli	Carrier of tendon stem cells supported TSPCs survival, proliferation, and metabolic activity over a long period of time and supported vascular-like structures	[140,174]
Carbodiimides	Cross-linker concentration	Pore size (SEM), cross-linking degree	Denaturation temperature, swelling, degradation	No	[117]
	Cross-linker concentration and steric bulkiness	N/A	Gelation time, tensile strength, elongation at break, denaturation temperature, degradation	Good adherence and spreading of corneal endothelial cells *in vitro*; successful mouse corneal transplantation model	[4]
Polyethylene glycol	Cross-linker concentration	Degree of cross-linking (free amino groups)	N/A	Differentiation of chondrocytes within hydrogel	[128]
		Fibril size (SEM, TEM)	Shear moduli, degradation	Fibroblast-seeded dermal equivalents showed greater viability in less cross-linked gels; cross-linked full-thickness skin equivalents are capable of recapitulating the morphology of human skin	[50]
	Cross-linker and collagen concentration, cross-linker branching	N/A	Shear moduli, compression moduli, swelling, degradation	Good non-cytotoxicity and attachment of fibroblasts in all tested conditions	[151]
	Collagen functionalization	Triple helix structure (circular dichroism analysis)	N/A	Endothelial cell and smooth muscle cell adhesion in hydrogels	[129]
Glycation	Cross-linker concentration	N/A	Compression moduli (confined)	No	[152]
		Fibril density (CRM)	Shear moduli	Glycated gels showed increased cell proliferation in the surface but decreased fibroblast invasion	[153]
		N/A	Circumferential tensile moduli, elongation at break, burst strength degradation	Survival of smooth muscle cells at 4 weeks in tunica media equivalents	[125,156]
		Fibril width, orientation (CRM)	Gelification time, turbidity, compression moduli	Viability, proliferation, and attachment of endothelial cells. Increased matrix stiffness enhances angiogenic endothelial cell spheroid outgrowth.	[157]
Genipin	Cross-linker concentration, cross-linking time	Fibril width (SEM)	Shear moduli, degradation, swelling	Greater MSC and NSC viability in less cross-linked gels	[161]
		Cross-linking degree (TNBSA)	Gelification rate, turbidity, shear moduli	MSC proliferation at all concentrations, as well as axonal growth in seeded dorsal root ganglia	[167]

The basic mechanical testing techniques used for polymeric materials, including hydrogels and collagen matrices in particular, include ​shear rheometry, (confined) compression, tension, or dynamic mechanical analysis [29]. Determining a value for the stiffness of the scaffold must be defined in terms of the type of deformation used by the technique (shear, compression, or tensile) and, consequently, comparisons should be made with caution in techniques using different deformation regimes. Collagen is a non-linear viscoelastic material; therefore, differences in the time-span or strain used during measurement acquisition can also influence the final value [8]. Swelling ratio measurements can also provide information about the nature, degree, and density of cross-linking in the polymer matrices ​and can be used to obtain indirectly the mechanical properties of the gel such as the Young modulus (E) [30]. A widely used molecular theory that describes the swelling of polymers in a solvent is the equilibrium swelling theory of Flory and Rehner, by which it is possible to associate swelling measurements with mesh size (ζ), degree of cross-linking ($\bar{MC}$), or elasticity modulus (E) of the hydrogel [31]. The theory, although initially developed for vinyl polymers and rubber-like materials, is applicable to fully swollen, isotropic networks, and in particular for biopolymers as demonstrated by Lin et al. [32], having been used in several systems with semiflexible chains, including collagen [[33], [34], [35]]. Furthermore, Marmorat et al. observed a good agreement of theoretically derived mesh sizes with cryo-SEM measurements for gelatin, a derivative of collagen [36].

Other models have been specifically used to computationally explain the mechanics of semiflexible polymer networks like collagen hydrogels ​and are able to predict the storage moduli of entangled or cross-linked collagen hydrogels from network parameters [[37], [38], [39]]. One of the most widely used models is the MacKintosh model [40,41], which considers biopolymer filamentous structures as worm-like chains, with a stretching modulus, μ, and bending modulus, κ. The storage modulus is predicted to scale as G′ ∼ κ^7/5^(c′·l)^11/5^, where κ is the bending modulus, c′ the concentration of filaments, and l the contour length of the chain [37,40]. Instead of constructing an analysis using artificially generated networks, Stein et al. developed a model from real network properties obtained from confocal microscopy, where the collagen fibers are considered elastic beams that resist stretching and bending and each cross-link is treated as a torsional spring [42]. By contrast, the Morse model also describes tightly entangled solutions of semiflexible polymers, but where the tangential flow of the filaments is assumed, allowing cross-links to break and rebuild instead of forming a fixed lattice [43].

The type of cross-linking can modulate the mechanical properties of collagen networks (and other semiflexible biopolymers) by imposing angular constraints on the cross-links, which modulate the flexibility of the biopolymer filaments. The extent of the constraint depends not only on the strength of the cross-linking bond ​but also on matrix topology i.e. mesh conformation [44]. In general, chemical cross-linkers will generate stronger covalent bonds, generating junctions that are fixed and less ‘floppy’ compared with other non-covalent binding forces. Adding chemical cross-linkers modifies the micro-mechanical response of collagen hydrogels under deformation, by limiting the slipping of physical cross-links between collagen fibers, and thus limiting stress relaxation typical of biopolymer networks [45]. Cross-linking mechanisms will also determine the viscoelastic behavior of the network. Mooney's group recently demonstrated how different combinations of ionic, physical, or covalent cross-linking in collagen-alginate hydrogels led to distinct viscoelasticity (evaluated through loss angle from shear rheology), which was correlated with a distinct expression of immunomodulatory paracrine markers in seeded MSCs [46]. In terms of the effects of cross-linking in the micro-architecture or pore size, this will also be determined by the type and degree of cross-linking. Zero-length and low-molecular-weight cross-linkers (Fig. 3) have a limited impact on changing the pore size of collagen networks, as they can only react with chemical groups that are already in close contact, changing the bending rigidity of collagen fibers (as predicted by the Mackintosh model) [47,48]. Cross-linkers with a significant chain length, like PEG, can impact the mesh size of nanoporous hydrogels ​but will not alter the pore size of micrometer-sized hydrogel networks [49,50]. The length or molecular weight of the cross-linker will also determine its ability to interconnect distant chains, having an influence on the degree of cross-linking, a parameter that has a deep impact on the mechanical properties of the hydrogel [19].

**Fig. 2:**
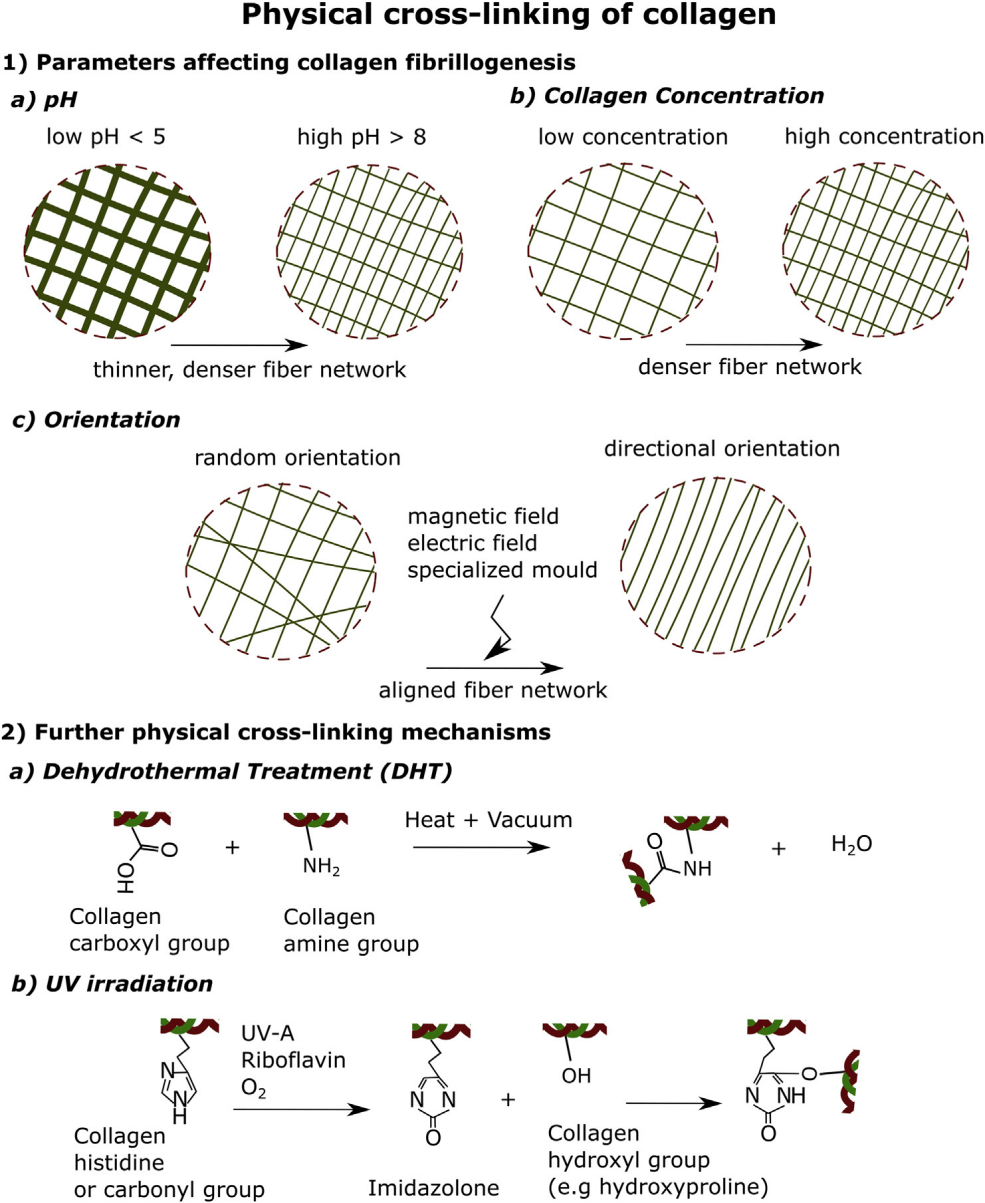
**Physical cross-linking mechanisms of collagen hydrogels.** 1: Showcases the parameters that affect collagen fibrillogenesis namely a) pH – collagen fibers become thinner and denser as pH increases b) collagen concentration – collagen fibers become denser as concentration increases and c) orientation – fibers can be organized directionally through magnetic and electric fields or a specialized mold. 2: Outlines the further physical cross-linking mechanisms used when designing collagen hydrogels. a) Dehydrothermal treatment (DHT) takes advantage of heat under vacuum to create amide bonds in collagen hydrogels and b) UV irradiation that binds histidines with hydroxyl groups.

**Fig. 3:**
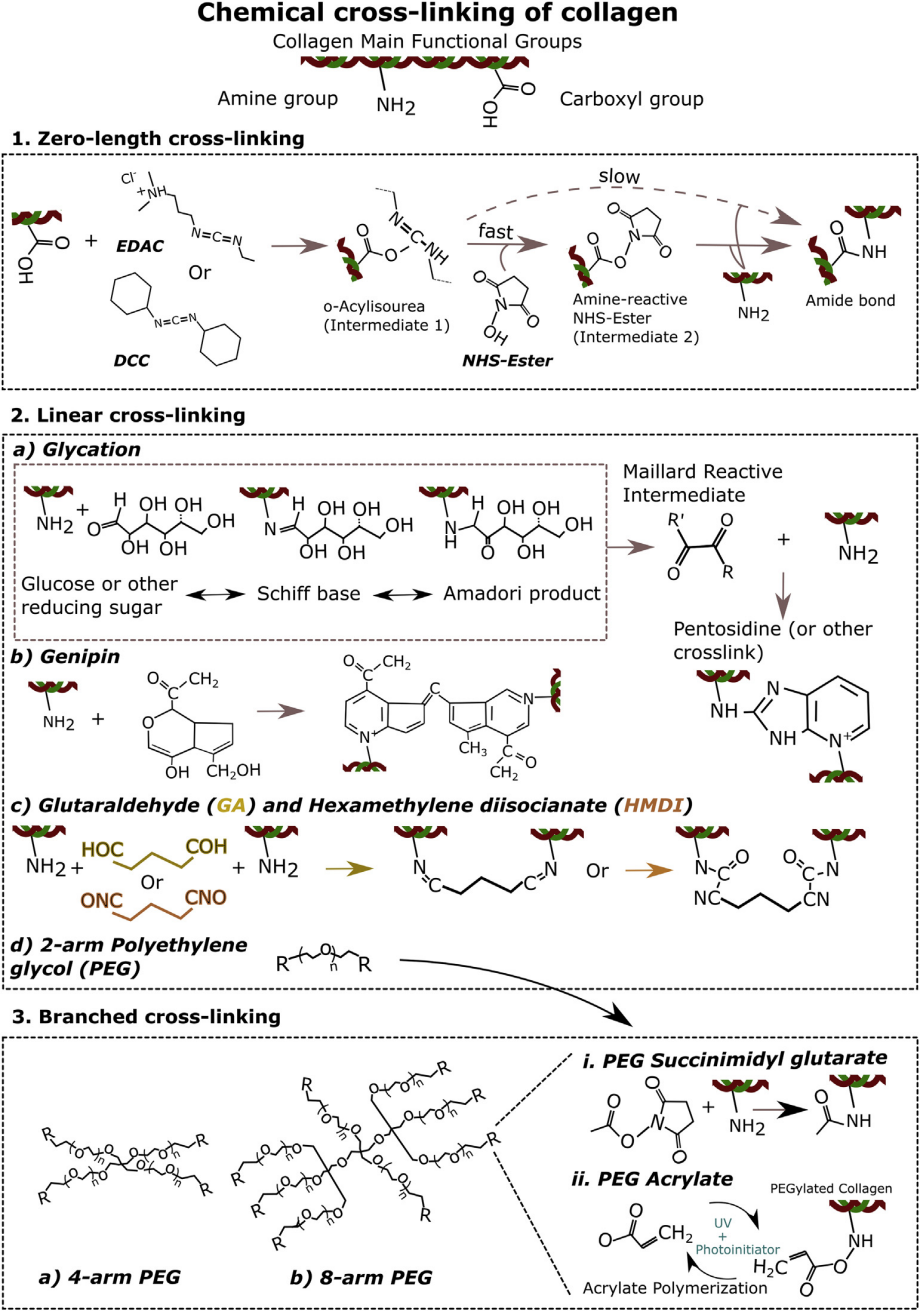
**Chemical cross-linking mechanisms of collagen hydrogels**. 1: Zero-length cross-linking using *N*-(3-Dimethylaminopropyl)-*N*′-ethylcarbodiimide (EDAC) or dicyclohexyl carbodiimide (DCC) as well as an N-hydroxysuccinimide (NHS)-Ester to form bonds between the carboxyl and amine groups of collagens. EDAC or DCC reacts with a carboxyl group to create a reactive intermediate (*o*-Acylisourea), which then reacts with an amine group to form a covalent peptidic bond. This reaction can be catalyzed by introducing an NHS–ester, which forms a more stable intermediate. 2: Linear cross-linking of collagen can occur through various reactions. a) Glycation, where a reducing sugar such as glucose interacts with an amine group to form a Schiff base. The complex reorganizes into an Amadori product and then a reactive intermediate, which can react with other amine groups to form complex cross-links such as pentosidine. b) Genipin interacts with amine groups and then forms cross-links by binding to adjacent-bound genipin molecules. c) Glutaraldehyde (GA) or hexamethylene (HDMI) bind amine groups on each side group of their carbon–carbon chain to form cross-links. d) PEG ​can be produced with 4 (depicted), 6, and 8 (depicted) arms (3.) to create branched cross-links, and its cross-linking chemistry will depend on the moieties of its end-groups. Some types of PEG used to cross-link collagen are PEG succinimidyl glutarate (3.i) which binds amine groups of collagen and PEG acrylate, which can bind to collagen PEGylated with PEG acrylate when using UV with a photoinitiator (3.ii).

### 2.1 Cross-linking mechanisms

#### 2.1.1 Physical cross-linking

Collagen I monomers self-assemble into fibrillar structures that may cross-link and/or entangle to form viscoelastic gels with varied network structures and mechanical properties in a process known as fibrillogenesis, that is generally accompanied by an increase in turbidity [51,52]. The fibrillogenesis curve has a sigmoid shape with a lag phase in which aggregation of collagen dimers and trimers in a linear-staggered with 4.4D periodicity occurs, accompanied by a growth phase where lateral aggregation of fibrils results in the abovementioned increase of turbidity [52,53].

The basic structure of collagen type 1 is modified at the posttranslational level, which can have an important effect on mechanical properties. An example of a posttranslational modification (PTM), which strengthens the collagen fiber is the hydroxylation of lysine residues within the collagen chains [54]. Hydroxylysine (hydroxylated lysine), together with telopeptidyl lysine, is essential in cross-linking tropocollagen molecules and collagen fibrils. Most hydroxylysine residues can be found in the collagen's telopeptides rather than the triple helix region [54]. The action of cross-linking is triggered by lysyl oxidase (LOX), which converts hydroxylysine and telopeptidyl lysine into hydroxyallysine and allysine (aldehyde) [55]. The spontaneous condensation of aldehydes with lysine and hydroxylysine form immature divalent cross-links which, in the body, mature into multivalent cross-links such as pyridinoline (PYD), deoxypyridinoline (DPD) [56], pyrrole [57] (bone), or dehydro-hydroxylysinonorleucine (deH-HLNL) (skin) [58]; the exact maturation process is complex and poorly understood [59]. Collagen hydrogels are manufactured with both atelocollagen (e.g. pepsin digested) and soluble telocollagen (e.g. acid-soluble). Acid-soluble telocollagen retains its telopeptides. Thus, tropocollagen molecules can self-assemble into fibrils, which form further cross-links with each other involving the lysine, hydroxylysine, and aldehyde residues mainly found within the telopeptides [58,60]. This stabilizes the collagen hydrogel and increases its mechanical properties [59]. On the other hand, pepsin-digested atelocollagen, which lacks telopeptides, forms fibrils and interfibrillar cross-links less effectively without the addition of non-collagenous molecules or further treatment [58].

Varying parameters including collagen concentration, ion content, anisotropy, isoelectric point (pI), pH, and temperature can alter gelation kinetics, stiffness, and the nanoscale fibrous architecture of the collagen hydrogels (Fig. 2). An excellent recompilation of the experimental studies that have used these parameters to modulate collagen hydrogel ​microstructure and mechanical properties can also be found in Antoine et al. [8]*.*

##### 2.1.1.1 pH and salt concentrations

Several studies have investigated the effects of changes in pH on the rate of fibril formation and morphology of collagen fibers [51,61]. The fibril diameter obtained from changes in pH ranges from 80 to 220 ​nm [51,61]. The fastest rates of fibrillogenesis occur between pH 6.9 and 9.2, with no significant changes in fibril diameters as seen in transmission electron microscopy (TEM), while gels formed at more acidic pH showed thicker fibrils and weaker strength [51]. Collagen has an isoelectric point (pI) of 9.3 in the absence of other electrolytes [51]. When pH approaches the pI, and at more basic pH, the surface charge of collagen monomers is reduced, resulting in increased hydrogen bonds between the triple helix. This minimizes the electrostatic repulsion between fibrils because of the increase of the triple helix stability, thus favoring collagen molecule ​aggregation [61,62].

It is generally recognized that collagen gels formed at a pH of 6 or lower have thicker fibers but are less densely packed than hydrogels formed at higher pH, being also softer. Such pH effect has been exploited for tissue engineering applications by several authors, after previous mechanical characterization with shear, tensile, or compressive tests showed higher moduli gels formed at slightly basic pH [[63], [64], [65]]. Yamamura and colleagues varied the stiffness of collagen gels of a fixed concentration by modifying the pH between 5 and 10 to study the formation of microvessel networks [66]. Relaxation modulus, as measured by uniaxial compression tests, was 4.6 times higher in their most alkaline composition compared with gels at pH 5, plateauing at 20 ​kPa ​at pH 8. Despite forming thinner fibrils in pH ​> ​8 hydrogels, these are more densely packed, increasing the relaxation moduli. Endothelial cell migration was hindered in rigid gels, but the formed microvessel networks were thicker and deeper [66]. A similar approach was undertaken by Chung et al., but gelifying 2 ​mg/ml type I collagen gels inside a specifically designed microfluidic platform for introducing vascular endothelial growth factor (VEGF) gradients, and varying the pH between 7 and 11. The low pH, soft collagen composition showed thick collagen fibers with large pore size, while the high pH collagen has thinner fibers and smaller pore size, inducing different angiogenic behaviors [67]. Roeder et al. characterized in more detail the effect of pH changes on the mechanical properties of collagen gels, and the relationship with their microstructural architecture, providing background to develop novel strategies for tissue repair [68]. Collagen hydrogels of 2 ​mg/ml polymerized at normal physiologic pH 7.4 had a linear modulus and failure stress of 16.6 and 6.0 ​kPa in uniaxial tension tests, respectively. In addition, matrices formed under increasingly acidic conditions showed a progressive decrease in mechanical properties, with basic gels being stiffer. Confocal microscopy showed how gel fibers under acidic conditions were not only thicker and less densely packed ​but also shorter compared with the stiffer basic gels.

Testing of different pH conditions has also been performed to evaluate the 3D-printability and suitability of fibroblast and keratinocyte-laden cells as skin engineering substitutes [69]. Collagen hydrogel scaffolds were printable at pH 5, 6, and 7, although only the latter was considered suitable for seeding with cells because it showed limited swelling. The gels showed good cell viability despite their low stiffness (20 ​Pa) measured by compression [69]. Nevertheless, there exists the concern that changing pH of the scaffold can affect cell viability if not maintained within the range 7.4–8.4 in applications involving cell encapsulation. We note that allowing cell invasion after the hydrogel is formed would involve degradation of collagen due to reduced pore size [10]. The ionic strength of the buffer used to dissolve collagen will also affect the final mechanical properties of the collagen hydrogels. Presence of different ions and their concentrations have a strong impact on the pI of collagen ​and will influence its net charge and the interactions between collagen chains [70]. Wood et al. already identified how increasing NaCl (thus ionic strength) in a NaOH–KH_2_PO_4_ buffer had a similar impact as decreasing the pH, by increasing the most frequent fibril size from 90 to 250 ​nm, although mechanical properties were not measured [51]. Changes in ionic strength lead to changes in fibril diameter, suggesting that lateral growth of collagen fibrils during fibrillogenesis also involves electrostatic interactions [71]. In terms of structural properties, increased ionic strength reduces the pore size ​and leads to a slower gelation kinetics [72]. Achilli et al. observed that increasing the ionic strength of collagen gels at specific conditions (pH 10 and polymerization temperature of 4 ​°C) could significantly increase the mechanical properties of the gel without affecting viability of cells seeded on top [64]. Typically, collagen hydrogel protocols use not only PBS10x as concentrated buffer ​but also others like (D)MEM10x, M19910x, and HBSS10x have been used [8]*.*

##### 2.1.1.2 Collagen concentration

Collagen concentration is another parameter that can easily be modified to tune hydrogels’ stiffness by increasing fiber density [73]. Mechanical properties increase in an inversely proportional way with concentration [37,45,63,74], except when under non-linear, high-stress deformation regime [47]. At the same time, an increase in collagen concentration does not result in an increase in fibril diameter [7,51,68] but in reducing the pore size of the fibrillary network [51,72]. Increasing concentration does not generally affect fibril diameter, which lie on the range between 270 and 290 ​nm [51,68,75,76]. Collagen concentration is an easily tunable parameter that has been used in a wealth of translational and *in vitro* studies.

Current studies tend to use collagen concentrations higher than 1 ​mg/ml as the resulting gels are easier to handle, can reach higher stiffness values and also for biological considerations, considering the concentrations found in native tissues. Rylander et al. produced gels of 4, 6, 8, and 10 ​mg/ml as these are concentrations commonly found in tissues *in vivo* [63]. In their study, they recognized the complex interplay between pH, concentration, and gelification temperature to obtain hydrogels of stiffness that can simulate different tissues, proposing an automated formula to determine, which conditions can produce a target modulus. The model was validated by matching collagen gels at pH 7.4 and 8 to match normal (2000 ​Pa) and cancerous (4000 ​Pa) human breast tissue both within their compressive moduli range between 540 ​Pa and 10,700 ​Pa and pore sizes from 1.2 to 3.2 ​μm [63]. Willits et al. developed gels between 0.4 and 2 ​mg/ml and measured their elastic moduli in a rheometer, obtaining 2.2 ​Pa for the gels prepared at the lowest concentration, and 17 ​Pa for the highest concentration. They found the maximum neurite growth from dorsal root ganglia (DRG) at the lowest collagen concentrations, so that the mechanical stiffness seemed to hinder neurite growth [77]. Overall, increasing the collagen concentration will result in a slight increase in strength, which can be amplified by adjusting other parameters like pH, but will also restrict cell migration and nutrient diffusion [15].

**Fig. 4:**
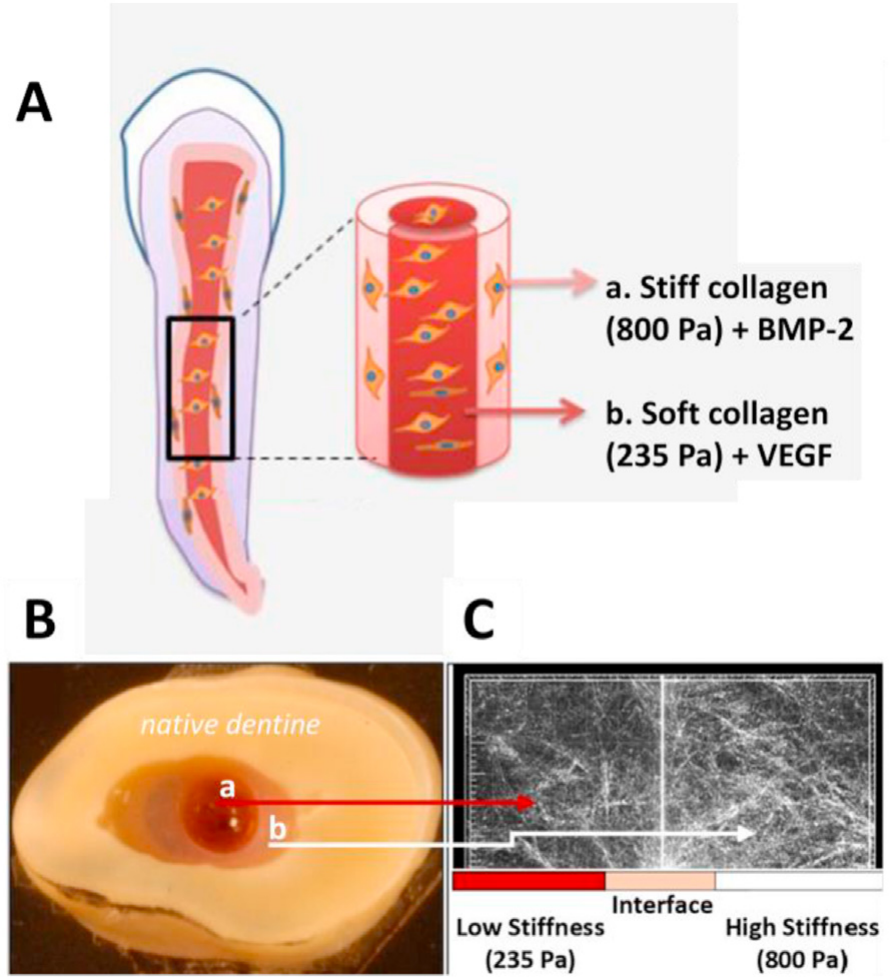
**Regeneration model for dental pulp canal using collagen hydrogel loaded with different growth factors:** A. Schematic illustration of model, with an outer collagen hydrogel of 2.88 ​mg/ml (800 ​Pa) containing dental pulp stem cells (DPSCs) and bone morphogenic protein 2 (BMP-2), and an inner collagen hydrogel (1.37 ​mg/ml, 235 ​Pa) containing DPSC and vascular endothelial growth factor (VEGF). B. Concentrically injected collagen hydrogels in tooth transversal slice. C. Interface adaptation between the two matrices evidenced by reflectance microscopy. Reproduced with permission from Ref. [78].

Pankajakshan et al. describe a modular collagen construct for dental pulp regeneration, consisting of central core of soft (285 ​Pa) collagen simulating the inner root canal, and an outer layer of stiffer (800 ​Pa) hydrogel interacting with the surrounding dentin. Stiffness was measured as shear storage moduli (G′) and varied by using a concentration of 1.37 ​mg/ml for the soft gel and 2.88 ​mg/ml for the stiffer one. VEGF ​was incorporated into the soft inner core and BMP-2 in the outer to foster endothelial proliferation/vasculogenesis and odontogenic differentiation of encapsulated dental progenitor stem cells, respectively. The combined effect of mechanical stimulation and GF delivery was further investigated *in vivo* (Fig. 4) [78]. Collagen concentration also has an influence in cell motility, as a result of the change in mesh size. A reference study by Saltzman and Parkhurst used collagen rat tail concentrations between 0.1 and 0.7 ​mg/ml to study neutrophil motility in collagen matrices. They first mathematically modeled cell motility was found to be near Brownian (random) motion inside the collagen gels ​and estimated the intercollagen spacing at 10 ​μm (approximately the size of a neutrophil) for their lowest concentration. In the experimental part, they identified an optimal concentration of 0.3 ​mg/ml for cell–fiber interactions with a peak in neutrophil motility, linearly decreasing at higher concentrations [79]. Also applied to cancer cell motility, Sapudom et al. studied the influence of pore size, fibril diameter, and elastic moduli in the capacity of cancer cells to invade and attach to collagen gels with concentrations 2, 2.5, and 3.5 ​mg/ml (smaller pore size and greater elastic moduli at higher concentrations) and also at different pH (which modifies the fibril size but not the elastic moduli) [74]. Interestingly, they found increased cancer cell invasion and cluster formation into the samples with greater fibril diameter ​but independent of the pore size and elastic moduli of the gels [74]. This highlights the importance of network properties and microscale mechanics and not just mechanical properties on cancel cell invasion of ECM-derived scaffolds.

##### 2.1.1.3 Temperature

Gelation temperature has a significant effect on polymerization kinetics, that will largely determine the mechanical properties of collagen hydrogels. Fibrillogenesis occurs faster at higher temperatures due to accelerated nucleation and lateral aggregation of collagen molecules [51,53]. With increased temperatures, collagen fibrils show smaller width [51,53] and are also shorter and randomly aligned [80,81], forming meshes with smaller, more homogenous pore size [63,80]. Lower temperatures are thought to limit nucleation of new fibers via decreasing entropy, which promotes thickening and elongation of already existing fibers, and forms networks, which are often more heterogeneous [82]. In terms of mechanical properties, trends are not so straightforward, with reports of increased stiffness for denser gels resulting from higher gelation temperatures (∼37 ​°C) [63], whereas other studies show greater compressive or shear moduli in gels produced at lower temperatures, e.g. 4 ​°C [64,83]. These discrepancies are thought to depend on pH and collagen concentration, and possibly on the mechanical testing setup [8,63].

Fischbach's group tested collagen hydrogels polymerized at 4, 20, and 37 ​°C to obtain gels of distinct microstructure and mechanical properties for myofibroblast differentiation and vasculogenesis [82,83]. In their first study, the authors verified experimentally and computationally that quick gelation at 37 ​°C yielded networks with thin fibers and small pores, whereas hydrogels at 4 ​°C showed thicker fibers and larger pores that were also stiffer under shear deformation [83]. They observe that the greater stiffness/larger pore size promotes cell contractility, local matrix remodeling, and changes in mechanosignalling stimulating ASC differentiation into proangiogenic myofibroblasts. The same hydrogels are tested in the second study with human cerebral microvascular endothelial cells and human umbilical vein endothelial cells (HUVECs) to evaluate microvessel formation. Hydrogels were polymerized at 4 or 37 ​°C and compared with the same compositions combined with Matrigel, a gelatinous ECM-derived material used in several biological applications [82]. Matrigel alters the microstructure of the hydrogels producing wider fibrils, and possibly larger pore size although this parameter was not measured. Fibronectin, laminin, entactin/nidogen-1, and perlecan found in Matrigel interfere with collagen fibrillogenesis by providing collagen nucleation sites [84]. Lumen formation was only observed in cold cast hydrogels mixed with Matrigel, and vascular networks were more branched in this composition, attributed to the microstructure triggered by gelation at 4 ​°C and the combination with Matrigel [82]. Achilli et al. evaluate temperature as one of the parameters tuned during hydrogel synthesis, also for vascular applications by seeding smooth muscle cells, a component of vascular structures [64]. The improvement of the mechanical properties of the gels prepared at 4 ​°C and at pH 10 was almost fourfold compared with the gels polymerized at 37 ​°C. Antoine et al. identified the reverse effect of gelation temperature on mechanical properties, with a significant positive correlation between temperature and stiffness, and a compressive modulus of up to 10 ​kPa using a collagen concentration of 10 ​mg/ml and pH 8.4 [63].

##### 2.1.1.4 Fiber orientation

Collagen is a fibrillar protein; therefore, matrix microstructural anisotropy and alignment can affect to a great degree its mechanical properties and cell organization [85]. In regeneration of certain tissues such as nerves and corneas, the alignment of collagen fibrils in hydrogels to guide cells and tissues is vital to regulate the migration, orientation, and shape of grafted cells [6]. Aligned constructs fail at lower strain but higher stress values than those with more random fibril organizations and consequently can bear more load. An example of this is tendon, whose aligned fibers shown tensile moduli of 43–1600 ​MPa whereas dermis, where collagen fibers show more random orientation, is 21–39 ​MPa [73]. Several methods have been reported to achieve collagen fiber orientation in hydrogels, including topography in microfabricated surfaces [86], magnetic flow or fields [20,[87], [88], [89]], electrochemical fabrication [90], continuous or cyclic stretching [85], extrusion and bioprinting [21,[91], [92], [93]].

Collagen hydrogels capacity for fibril alignment and tensile properties have been exploited in muscle and vascular regeneration applications. Cummings et al. produced aligned collagen vascular constructs of high stiffness by cyclic straining, with a tensile modulus of 200–250 ​kPa that allowed endothelial cell proliferation [94]. Another study reported an 80% increase in tensile Young's modulus (13.4 ​± ​9.6 ​kPa) compared with measurements along the perpendicular direction in myoblast-seeded collagen gels aligned by acoustic patterning [95]. The authors later showed how collagen effectively maintained the viability of the cells as they shifted from a rounded morphology into adherent myoblasts, which contracted the aligned gels while retaining the patterned configuration. Brookes et al. fabricated tissue-engineered aligned muscle constructs by polymerizing rat-tail collagen type I encapsulating C2C12 and muscle progenitor cells inside a cylinder under constant flow [92]. The constructs showed fibrillary and cell alignment in comparison with standard polymerization methods. The authors found more extensive myotube formation for constructs prepared at high cell densities (10^7^ ​cells/mL) within low stiffness matrices (200 ​Pa) and investigated it further in laryngeal rat partial laryngectomy models.

Fiber alignment is especially critical in some specific applications like nerve regeneration, and collagen has enabled significant progress in the field. In very early studies, Tranquillo's group described how magnetically aligned collagen gels, thanks to the diamagnetism of the peptidic bond initially described in 1984 [96], stimulated and directed neurite elongation and Schwann cell invasion in DRG [20,88]. More recently, Antman-Passig et al. produced mechanical orientation of collagen gels by uniaxial cyclic stretching using glass capillaries [85]. The gels had a comparatively low mechanical strength (shear moduli of 140 ​± ​30 ​Pa), but fibrillar orientation was enough to induce increased neurite elongation in DRG, primary neurons, and neuron-like pheochromocytoma PC12 ​cells in comparison with randomly aligned gels. Orientation of collagen fibrils is also crucial for corneal regeneration. Kim et al. printed in an extrusion-based bioprinter collagen seeded with keratinocytes with needles of different diameter. In the smallest diameters tested (25 and 30 gauge), for which greater shear forces were applied, aligned cells were observed along the direction of the printing path ​and had higher expression levels of the keratocyte-specific genes and keratocan than those of the other groups. Keratocytes also produced more collagen I in the aligned gels, indicating that collagen fibrils provide a stroma-like environment. Matrix remodeling was also associated with 45% higher transparency compared with non-aligned control hydrogels [97].

A special case of collagen alignment procedure is plastic compression, which consists on subjecting dilute collagen hydrogels to known loads, expelling >80% water from the gel and aligning fibers in the direction of water removal [98,99]. Plastic compression causes fiber densification and significantly increases the mechanical properties of gels without inducing significant cell death, and has been used to engineer skin substitutes [100], artificial corneas [101], articular cartilage [102], or artificial laryngeal cartilage [103].

##### 2.1.1.5 UV cross-linking and dehydrothermal treatment

Collagen can be cross-linked using other physical methods that do not take place naturally *in vitro*, by using external physical factors that induce chemical cross-links between collagen fibers. Although UV cross-linking and DHT ​induce partial collagen denaturation, they are successful in modulating the mechanical properties and microstructural organization of collagen hydrogels. DHT is a cross-linking method known since the 1960s, where collagen carboxylic acid and amino side chains are cross-linked via condensation in a vacuum oven [104]. DHT is more commonly used in membranes, fibers, and scaffolds as it involves complete drying of the samples subjecting to temperatures of >90 ​°C, [[105], [106], [107], [108]]. UV cross-linking produces free radicals on tyrosine and phenylalanine residues that cross-link collagen [109]. It is much faster than DHT, reducing cross-linking time from days to minutes [110]. Irradiation doses up to 500 ​Gy have been reported to increase storage modulus up almost 150% [111], nevertheless other authors have documented a reduction of stiffness with increased UV irradiation time, possibly associated with degradation of collagen matrix [109]. In the same study, the combined use of UV cross-linking and DHT showed increased mechanical properties while cell proliferation was not negatively affected. Riboflavin, a photosensitizer that generates a singlet oxygen, has been used to aid photo cross-linking of collagen and tested for meniscus tissue engineering applications [112]. Adjuvant use of riboflavin in UV cross-linking increases the Young's modulus of 3.5 ​mg/ml rat tail collagen hydrogels from 150 to 600 ​kPa with 45–60 ​min irradiation, while hydrogels that do not contain riboflavin are degraded with increased irradiation time [113]. The UV/riboflavin cross-linking procedure is actually used by ophthalmologists in a procedure called corneal cross-linking for the treatment of ketatoconus, a condition in which the central area of the cornea becomes thinned [114].

#### 2.1.2 Chemical and enzymatic cross-linking

Covalent cross-linking of collagen hydrogels is commonly used in drug delivery and tissue engineering applications to control *in vivo* absorption of collagen and to increase the mechanical properties of the material. In physiologic conditions, stabilization of fibrils is reinforced by means of cross-linking by condensation of lysine and hydroxylysine residues and aldehyde formation, a reaction catalyzed by the enzyme lysyl oxidase. This mechanism provides the collagen fibrils with higher tensile strength, necessary for tissue integrity [115,116].

In the introduction of this section, we have already described how different type of cross-linkers can impose angular constraints in the cross-links reducing the flexibility of the collagen fibers [44]. Some of the most commonly used cross-linking agents are glutaraldehyde, carbodiimides (e.g. ​1-ethyl-3-(3-dimethylaminopropyl) carbodiimide – EDAC). [117,118], chromium tanning [119], formaldehyde [120], polyepoxy compounds [121], acyl azide [122], hexamethylene diisocyanate (HMDI) [123,124] carbohydrates e.g. (ribose [125], glucose [126]), and plant extracts, especially genipin. Many of these cross-linking methods have also been reviewed for collagen fibers but not hydrogels [127]. Recently, the influence of polyethylene glycol (PEG) polymers that can vary in molecular weight, degree of branching, and terminal groups have been intensively evaluated for cross-linking and functionalization of collagen-based devices [126,[128], [129], [130]] (Fig. 3). Branched cross-linkers increase the number of fibers that meet at each network junction, known as local connectivity (z) of the network, contributing also to the mechanical stability of the hydrogel [39].

##### 2.1.2.1 Glutaraldehyde

Glutaraldehyde (CHO(CH_2_)_3_CHO) is a bifunctional cross-linking agent, which forms monomeric or oligomeric covalent bonds between two amino acid side chains, typically lysyl and hydroxylysil residues within collagen [131]. Glutaraldehyde and its shorter-form formaldehyde (HCHO) are extensively used as fixation agents for bacteria, cells, or tissues [132], but they are far from ideal for hydrogel cross-linking because their bonds are transient and sustained release of monomers over time is cytotoxic [133]. Glutaraldehyde at low concentrations was among the first studied cross-linkers for collagen ​and for scaffold cross-linking [131,134,135]. Nowadays, however, glutaraldehyde is mainly used as control cross-linking molecule, to compare the degree of cross-linking or amount of free amines with respect to other cross-linking methods, due to the fact that glutaraldehyde is an active cross-linker that can react with virtually any amine group in collagen [118].

##### 2.1.2.2 Hexamethylene diisocyanate

Isocyanates are another versatile cross-linkers for collagen hydrogels. HMDI ​was initially studied as a possible alternative to glutaraldehyde to cross-link collagen scaffolds in a less cytotoxic procedure [136]. Reaction between the isocyanates in HMDI and amines in the collagen molecule generates cross-links containing stable urea groups [137]. The low solubility of some isocyanates in water means a surfactant is needed to promote the reaction with free amine groups. Isocyanates are strong, linear cross-linking agents that can significantly increase the mechanical properties of collagen biomaterials, including microspheres [123] hydrogels [137], scaffolds [138], membranes [139], or fibers [127]. Butane diisocyanate has been used as functional group with pluronics to generate a thermosensitive collagen-butane diisocyanate hydrogel for tendon stem cell delivery, which showed reduced contraction compared with untreated gels [140]. Currently, the commercially available Collagen Repair Patch from Zimmer ​uses a proprietary isocyanate cross-linking technique [3].

##### 2.1.2.3 Carbodiimides

Carbodiimides such as cyanamide or 1-ethyl-3-(3-dimethyl aminopropyl) carbodiimide hydrochloride (EDAC or EDC) are alternative, widely used zero-length cross-linkers that can establish peptide-like bonds between carboxyl and amino groups in collagen without becoming a final part of the amine bond [133]. The by-product of the reaction is a non-toxic chemical, urea ​and can be washed away after cross-linking [141]. Cross-linking with EDAC is also attractive for biomedical applications as it can take place on physiological-like conditions (pH 7.4, 37 ​°C), although its maximum efficiency is at a mildly acidic pH (4.5). Another important consideration is that although it is compatible with phosphate buffers, amine containing buffers (e.g. ​Tris) should be avoided as they can quench the reaction [142]. NHS ​is often used with EDAC to improve efficiency of the reaction by creating dry-stable intermediates.

EDAC has been extensively evaluated as a cross-linker molecule for biomedical applications. Yang et al. showed how increasing the concentration of EDAC reduces swelling ratio and increases resistance to enzymatic degradation in 2 ​mg/ml collagen hydrogels [117]. Authors report no negative effect in cell adhesion and proliferation. Although EDAC/NHS is largely considered a biocompatible cross-linking method, some authors have noted the possible negative effect of reducing the availability of the carboxylate anion of glutamic acid, which is critical for cell attachment to collagen. Cross-linking with EDAC/NHS was shown to modulate both the affinity and the mode of cell interaction with collagen films, resulting in decreased cellular spreading, survival, and growth [143]. This can be understood within a general discussion about using or not cross-linkers, as most of them intervene with cell-adhesive motifs [144]. Vogel et al. compared non-cross-linked and EDAC-cross-linked collagen hydrogels with a stiffness of 90 and 160 ​Pa, respectively, to assess the osteogenic differentiation of MSCs, which was found for both conditions after 22 days of culture [145]. Another carbodiimide, N-Cyclohexyl-N′-(2-morpholinoethyl) carbodiimide metho-*p*-toluenesulfonate (CMC), was evaluated as a substitute for EDAC for corneal applications [4]. CMC contains two heterocyclic compounds, which reduce mobility of the molecule and increase its steric hindrance. CMC/NHS-cross-linked collagen hydrogels exhibit superior tensile strength, closer to that of human cornea, and decreased elasticity. CMC hydrogels were also more resistant to collagenase degradation. Both EDAC and CMC cross-linked hydrogels showed similar corneal epithelial cell proliferation after 15 days. Carbodiimide chemistry can also be used to couple collagen with other carboxyl-containing molecules like hyaluronic acid [146,147].

##### 2.1.2.4 Polyethylene glycol

PEG ​has been extensively used as a matrix for controlled drug and cell delivery, owing to its biocompatibility, non-immunogenicity, low protein adsorption, and long history of safe *in vivo* use, as well as the versatility of its chemistry [14,148]. PEG resists recognition by the immune system, and its rapid clearance from the body has permitted FDA approval for several biomedical applications [149]. PEG can have linear or branched structures, and its basic form has end hydroxyl (-OH) groups, which can be functionalized with other groups (e.g. acrylates, azides, maleimide) for different kinds of cross-linking [148]. An improved utilization of PEG is through star polymer structures: three-dimensional hyperbranched structures in which linear arms of different molecular weights stem from a central core. Star polymers may be used in a variety of biomedical applications because they provide a high density of functional groups in a small volume ​and are thus attractive as drug delivery carriers [150].

PEG in its various forms enables enhanced tunability for mechanical optimization of the collagen gels, without affecting cell viability. Branched PEG can have a more radical effect on the mechanical properties even at low concentrations. In a thorough study, Lotz et al. used four-arm polyethylene glycol succinimidyl glutarate (PEG-SG) at two different cross-linking ratios, 50% and 100%, obtaining an Young's-modulus of 557 and 1225 ​Pa, respectively, while the non-cross-linked control did not surpass 320 ​Pa [50]. Collagen hydrogels exhibited fast degradation on collagenase digestion, whereas semi-cross-linked PEG-SG maintained 35% of the initial mass, which was increased up to 60% for fully cross-linked hydrogels. In terms of microstructure and pore size, the fully cross-linked gel led to hydrogels with a visibly denser network in comparison with semi-cross-linked gels, and no fibril-like structure formation. Both 50 and 100% cross-linked hydrogels showed inhibited shrinkage in comparison with native fibrillar collagen gels. Finally, all *in vitro* skin models based on both cross-linked and non-cross-linked hydrogels formed a multilayered epidermis and similarities to human skin. Sargeant et al. compared cross-linking collagen four-arm to eight-arm PEG SG, which is expected to further decrease the pore size and increase the stiffness of the hydrogels. Indeed, the compressive modulus was significantly higher for the eight-arm PEG formulations compared with those with four-arm PEG-SG, reaching 20 ​kPa [151]. Overall, the hydrogels composed of eight-arm PEG exhibited minimal swelling/shrinkage (less than ± 5 ​wt.%), while the hydrogels composed of four-arm PEG swelled in excess of 100 ​wt.%. It is worth noting though that neither the molecular weight nor the collagen concentration (which was in the high 50–100 ​mg/ml range) were kept constant in both conditions, which may affect interpretation of the results. The gels were equally non-cytotoxic and allowed fibroblast proliferation. Taguchi et al. evaluated as well the cytotoxicity of PEG-SG cross-linker with an alkalized collagen gel prepared at a 4S-PEG concentration of 1 ​mM for cell encapsulation by histology and 3-(4,5-dimethylthiazol-2-yl)-2,5-diphenyltetrazolium bromide (MTT) cytotoxic assay after 2 days [128]. Other functionalizations and degrees of branching have been used based on the PEG molecule. Cosgriff et al. tested PEG-diacrylate cross-linker for the stabilization of bacterial collagen hydrogels. The presence of acrylates allows for photo-cross-linking with an appropriate photoinitiator. The authors showed that the PEG conferred much of the mechanical strength to the hydrogels, obtaining compressive module values of around 140 ​kPa ​at 6 ​mg/ml of collagen concentration [129].

##### 2.1.2.5 Glycation

Non-enzymatic glycation has also gathered interest as a cross-linking method for collagen [152,153]. Collagen cross-linking by glycation or glycosylation is a pathologic process occurring *in vivo*, accelerated in mammalians during aging or in conditions like diabetes. Reducing sugars covalently bind to free amine groups and establish cross-links with ECM proteins and with other glucose-modified proteins (the so-called advanced glycation end-products) [154,155]. An advantageous characteristic of glycosylation as a cross-linking mechanism is its capacity to alter the mechanical properties of collagen gels with non-toxic molecules like glucose or ribose. Increasing concentration of glucose-6-phosphate (G6P) has been shown to reduce degradability of collagen hydrogels by collagenase without affecting cell viability [156]. Incubation of collagen with G6P has also been shown to inhibit gel contraction by cells in a concentration-dependent manner [155].

Glycosylation has therefore been studied as a potential cross-linking method for plenty of tissue engineering applications. Roy et al. used collagen type I hydrogels pre-incubated with 250 ​mM ribose, showing a 10-fold increase in bulk modulus with respect to untreated collagen gels, to encapsulate chondrocytes [152]. The authors found increased glycosaminoglycan (GAG) and collagen endogenous production in comparison with standard collagen gels after 4 weeks. In early studies, Tranquillo's lab reported that incubation of collagen type I tunica-media coronary equivalents with 30 ​mM ribose for 10 weeks increased the circumferential tensile strength and moduli with respect to controls [125]. Encapsulated smooth muscle cells induced circumferential alignment of collagen fibers, which was necessary together with glycation-based cross-linking to produce matrix stiffening. As a result, the vascular equivalents closely recapitulated the non-linear stress–strain curves of a rat aorta. Another study by Mason et al. reported an increase of compressive moduli in 1.5 ​mg/ml gels from 200 ​Pa in the non-glycated gel to 700 ​Pa in the gels incubated with 250 ​mM ribose [157]. The authors also showed that endothelial cells (ECs) remain viable and proliferate after 3 weeks of culture, showing that glycation does not alter EC viability and proliferative potential. In addition, endothelial cell spheroids cultured on the stiff, glycated gels showed a twofold increase in the total extension length per spheroid and a 1.5-fold increase in the average number of extensions. The participation of glycation cross-linking in pathologies like diabetes or in tumor progression [158], together with the documented effects of advanced glycation end-products on stem cell turnover [159] may limit its applicability for tissue engineering applications, and more studies are needed to dilucidate its effect on cell adhesion and influence on phenotype expression.

##### 2.1.2.6 Genipin

Several researchers have evaluated other non-cytotoxic molecules for cross-linking of collagen, finding in natural-plant extracts like genipin [[160], [161], [162]], procyanidin [163], or oleuropein [164] some attractive candidates. Among them, genipin, a natural cross-linking agent derived from the gardenia fruit, has been among the most studied [165]. Genipin reacts non-specifically with primary amine groups to produce a secondary activated form of genipin, and its ester group forms a secondary amide bond with proteins [166]. The oxygen radical-induced polymerization of genipin releases blue non-toxic pigments that caused the gels to assume a blue color. Genipin itself is significantly less cytotoxic than glutaraldehyde, although this is concentration-dependent [165]. In tissue engineering applications, Macaya et al. verified that genipin collagen gels possess a high resistance to collagenase degradation, nevertheless neural stem cell (NSC) viability after 24 ​h was decreased up to 60% with 0.25 ​mM of cross-linker and was as low as 5% for 0.5 ​mM [161]. Výborný et al. compared genipin with EDAC cross-linking in ECM-based hydrogels (composed mostly of type I collagen) ​and observed that at the same concentration, genipin had higher cross-linking capacity (up to 50% using 10 ​mM) and also led to stiffer gels as measured by rheology (G’ of 100 ​Pa compared with 20 ​Pa) [167]. Genipin also led to gels less liable to contraction caused by seeded cells. Cross-linking the hydrogels with a concentration of 5 ​mM of both cross-linkers significantly decreased MSC proliferation attributed to cytotoxicity of unbound cross-linker molecules, while a smaller concentration of 1 ​mM did not affect proliferation in comparison with non-cross-linked hydrogels. Such concentration did not seem to negatively affect NSC differentiation in encapsulated DRGs ​and was further evaluated in *in vivo* studies [167].

##### 2.1.2.7 Transglutaminase and other enzymes

Enzymatic cross-linking with transglutaminase or LOX ​are other cross-linking methods that create bonds, which are compatible with cells and naturally present *in vivo*. Transglutaminases can form an isopeptidic bond between glutamine and lysine from different proteins, and its resulting bonds are highly resistant to proteolysis [168]. A more in-depth review focusing on transglutaminase cross-linking for collagen and other proteins has been published [169]. Lee et al. showed that stiffer hydrated collagen matrices cross-linked with 500 ​μg/ml of transglutaminase result in increased endothelial sprouting and obtained lumen-like structures in a vasculogenesis study [170]. Transglutaminase has been used as a cross-linker for collagen with a wide range of stiffness for developing tumor *in vitro* models with unique properties [171]. LOX oxidatively deaminates lysine and hydroxylysine residues in the telopeptide domains of collagen, enabling the formation of cross-links between collagen fibers to form a 4D staggering [172]. A drawback of transglutaminase and LOX cross-linking is that they have a limited effect on the mechanical properties, and in addition, they are cost prohibitive for large-scale or clinical applications [173].

## 3 Importance of growth factor loading in collagen-based scaffolds

GFs ​are powerful molecules involved in various cellular processes and often function as the signaling molecules between cells. They cause cell proliferation, maturation and, at times, differentiation, making these molecules particularly important for tissue regeneration [12]. The challenge in using GFs in tissue engineering is to keep them active and stable within the material for a prolonged period to allow cells to migrate to the site of injury, proliferate, and differentiate. This requires GF binding sites that can immobilize, stabilize, and present the GF to cells in a useful manner as well as release it in a more timely manner into the environment [12].

**Fig. 5:**
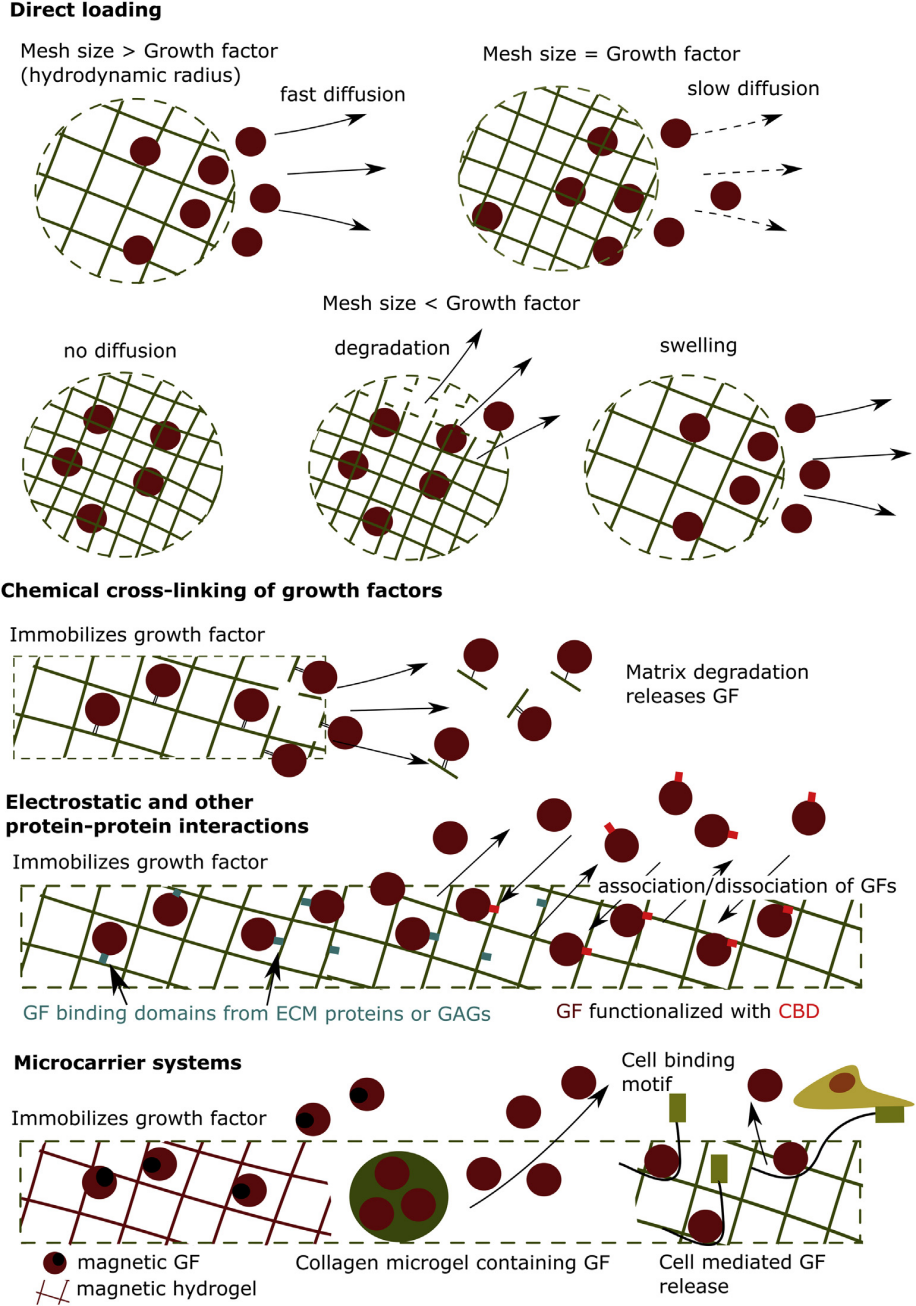
**Collagen hydrogel ​systems designed to deliver growth factor (GF) in situ:** The systems vary from simple where GF is directly loaded into the hydrogel and released by quickly diffusing into the extracellular space, to chemically cross-linking the GF to the hydrogel, which limits release but can impede its bioactivity. More complex, carrier delivery systems and hydrogel designs can find a balance between the two extremes and release GF in a sustained manner without affecting its bioactivity. CBD: collagen binding domain.

Collagen type I, a material often used in tissue engineering, does not have a high affinity and binding capacity to GFs [175], thus different strategies have been explored to retain the GFs in scaffolds based on this natural material (Table 2) (Fig. 5).

**Table 2:** **Collagen–growth factor (GF) release systems:** summary of their growth factor retention ability and their main benefit.

Mechanism of GF loading	GF system used	GF remaining in system	*In vivo* and *in vitro* effect	Other findings	References
Direct loading (No cross-linking or other modifications)	bFGF	50% after 7 ​d	N/A	The affinity of bFGF to collagen was examined and compared with other GFs.	[177]
		15–30% after 7 ​d	N/A	Collagen was found to function as a bFGF reservoir *in vivo*	[178]
		40–80% after 70 ​h	N/A	Collagen was found to function as a bFGF reservoir *in vivo* and *in vitro*.	[178]
		45% after 7 ​d	Dual release of HGF and bFGF from collagen enhanced blood vessel formation.	N/A	[108]
	HGF	30% after 7 ​d	N/A	The affinity of HGF to collagen was examined and compared with other GFs.	[177]
		35% after 7 ​d	Dual release of HGF and bFGF from collagen enhanced blood vessel formation.		[108]
	PDGF-BB	30% after 7 ​d	N/A	The affinity of PDGF-BB to collagen was examined and compared with other GFs.	[177]
	VEGF	<15% after 7 ​d	N/A	The affinity of VEGF to collagen was examined and compared with other GFs.	[177]
	IGF-1	<5% after 7 ​d	N/A	The affinity of IGF-1 to collagen was examined and compared with other GFs.	[177]
	HB-EGF	<5% after 7 ​d	N/A	The affinity of H -EGF to collagen was examined and compared with other GFs.	[177]
	rh-BMP2	N/A	N/A	Certain isotypes of rh-BMP2 have a pH- and salt-dependent increase in affinity for collagen.	[179]
Chemical cross-linking	VEGF bound to collagen using EDAC	50–70% depending on cross-linking concentration compared with <10% for the non- cross-linked system at time point 0 ​h.	Increase in viability, invasion and assembly of endothelial cells into the collagen hydrogel compared with the no VEGF and soluble VEGF groups.	N/A	[193]
	EGF PEGylated with PEG-NHS and bound to collagen	N/A	Cell proliferation of cross-linked EGF using PEG-NHS was lower compared with the non- cross-linked condition due to PEG sterically hindering cells of attaching to the EGF properly.	Cell proliferation was highly dependent on the site of EGF PEGylation. PEGylation at the N-terminus showed the best, albeit still lower than non- cross-linked EGF, and PEGylation at Lysine 48 the worst biological response *in vitro*.	[194]
	EGF cross-linked using riboflavin	96–98% after 120 ​h	Cytokeratin (CK) 3/12 – an important corneal epithelial cell differentiation marker – was upregulated and tight junction were observed between adjacent cells.	No significant difference was observed between the immobilized EGF and the soluble EGF group in terms of differentiation	[195]
Electrostatic and other protein–protein interactions	rhFGF-2 interacting with heparan sulfate bound to collagen	60% in hydrogels with heparan sulfate and 20% without after 21 ​d	The collagen-HS-bFGF complex showed extensive angiogenesis throughout the hydrogels *in vivo*, which was not the case in the collagen-bFGF and collagen-HS hydrogels.	N/A	[197]
	EGF interacting with hyaluronan bound to collagen	N/A	Aided in keratinocyte migration in a scratch assay as well as EGF-signaling and HGF expression of fibroblast, which affect keratinocyte differentiation. HA-EGF containing hydrogels also resulted in more effective wound healing compared with the no-EGF group.	Sulfated hyaluronan increased EGF binding to collagen compared with heparan sulfate and hyaluronan	[198]
	bFGF bound to heparinized collagen	60% in hydrogels with heparan suldate and 20% without after 250 ​h	N/A	N/A	[199]
	EGF expressed with a collagen binding domain (CBD) bound to collagen	N/A	Gene expression analysis also revealed that neural stem cells in the EGF-CBD-Collagen expressed significantly more stem cell, neuron, astrocyte and oligodendrocyte associated markers compared with unbound EGF. Cell proliferation was also increased in the former.	N/A	[175]
	VEG121 merged with Fibronectin Collagen Binding Domain (FNCBD) bound to collagen	N/A	FNCBD-VEGF121 showed a similar bioactivity to soluble VEGF121 but significantly increased the expression of VEGFR-2, a receptor for VEGF – on endothelial progenitor cells (EPCs). *In vivo* experiments showed that the novel chimeric growth factor could induce EPC mobilization locally without having a system effect on the cell type.	N/A	[200]
Other carrier Systems	Collagen microgels containing rhBMP-2.	80% BMP-2 retained after 14 ​d			[123,201,202]
	EDAC-NHS cross-linked Collagen microgels containing rhVEGF	rhVEGF was released in 8 days in collagenase and in 4 weeks in cell medium.	HUVEC cultures that show capillary formation after 21 days, comparable with a control with VEGF in solution.	N/A	[203]
	Magnetic GFs	N/A	The study showed that scaffolds supported cell adhesion and proliferation.	Magnetic nanoparticles did not leak out of the scaffold over time.	[204]
	VEGF and PDGF-BB immobilized using the TrAP system	<1 ​ng/ml release with TrAP over 49 ​h compared with 3 ​ng/ml without TrAP	TrAP-PDGF-BB functionalised coverslips resulted in increased cell proliferation compared with soluble PDGF-BB in 2D. There was no significant difference between collagen hydrogels loaded with PDGF-BB and TrAP-PDGF-BB decorated collagen hydrogels in 3D	N/A	[205]

### 3.1 Direct loading

The simplest way to incorporate GFs into a collagen hydrogel system is to incorporate them directly into the collagen matrix. If no modification is made, then the release – governed by diffusion – will likely show a rapid burst at the start with most of the GF leaking out of the material [176]. Kanematsu et al. [177] developed a collagen-based hydrogel formed in solutions of basic fibroblast growth factor (bFGF), hepatocyte growth factor (HGF), platelet-derived growth factor (PDGF-BB), VEGF, insulin-like growth factor-1 (IGF-1), and heparin binding epidermal growth factor-like growth factor (HB-EGF). VEGF, HB-EGF, and IGF-1 exhibited the characteristic burst release profile and substantial loss of loaded GF. However, HGF, bFGF, and PDGF-BB showed a somewhat sustained release profiles, which were parallel to the biodegradation profile of the collagen matrix. Thus, depending on the GF collagen can function as reservoir suggesting that direct loading should not be discounted as a strategy depending on the GF and application parameters [177,178].

Collagen–GF interaction can also be enhanced in a pH-dependent manner. Studies with recombinant human bone morphogenic protein 2 (rhBMP-2) showed that rhBMP-2 binding to collagen could be enhanced not only by increasing the pH but also by adding NaCl to the carrier solution. Proton ions likely facilitate the self-association of rhBMP-2 to form layer on collagen. Thus, rhBMP-2 could be loaded more efficiently into collagen hydrogels resulting in improved osteoinductive materials [179].

Collagen–GF interaction is mostly weak, and the release profile of proteins from the hydrogel matrix is governed by diffusion and steric hindrance. Hydrogel mesh size can range from the nanometer- [180] to the micrometer [177]-scale depending on collagen type used, cross-linking concentrations, temperature, and pH during the hydrogel production process [181]. When the mesh size of the hydrogel is greater than the GF/molecule loaded, then the release is primarily affected by diffusion, especially at low GF concentrations. Diffusion is a major mechanism of GF release from highly porous fibrillar collagen hydrogels, which are commonly used in tissue engineering. When a GF/molecule is loaded, particularly when it has low affinity for collagen, rapid release is observed [182]. This lead researchers to use high concentrations of GF e.g. BMP-2 to achieve the desired effect i.e. bone lesion closure but can cause side-effects such as ectopic bone formation [182]. As GF concentration increases, the hydrogel can become ‘crowded’, and steric hindrance could play an increasing role [63]. If there is increased GF/molecule affinity to the collagen matrix, as Kanematsu et al. [177] described for some GFs, the release cannot be described by simple diffusion, but parameters such as the collagen–GF interaction strength and the degradation rate of the collagen matrix bound to the GF need to be taken into account. These are discussed in later sections of the article.

The diffusivity of proteins from a hydrogel follows the Stokes–Einstein equation for diffusion of spherical particles in a fluid:

$$D\;=\;\left(k_{B}T\right)/\left(6\pi \eta R_{Η}\right)$$

D ​= ​diffusivity, k_B_ ​= ​Boltzmann's constant, T ​= ​temperature, η ​= ​kinematic viscosity of fluid, R_H_ ​= ​hydrodynamic radius.

As the R_H_ increases at constant T, the diffusivity of the protein out of the hydrogel decreases. Apart from size/weight, R_H_ also depends on pH, which can greatly influence the number of water molecules attracted to the protein diffusing through the hydrogel [63].

Equations based on Fick's law of diffusion can be used to calculate the ratio of the amount of molecule released at time t to the total amount that can be released Mt/M∞:

$$\frac{M_{t}}{M_{\infty }}\;=\;1-\;\sum_{n=0}^{\infty }\frac{8}{{\left(2n+1\right)}^{2}\pi^{2}}e^{\left[\frac{-{\left(2n+1\right)}^{2}\pi^{2}Dt}{L^{2}}\right]}$$

D ​= ​diffusivity, L ​= ​half-thickness of hydrogel.

This equation although straightforward is based on the assumption that the diffusing molecule is spherical [183]. A simpler empirical equation developed by Peppas et al. can also be used [184,185]:

$$\frac{M_{t}}{M_{\infty }}\;=\;kt^{n}$$

k ​= ​constant that depends on structure and geometry, n ​= ​release exponent depending on geometry (e.g. cylinder n ​= ​0.45).

However, these empirical models can only predict a release profile once experiments have been conducted and are not accurate once the system has been changed in some capacity e.g. cross-linker increased. It is also important to note that release models change according to the geometrical parameters of the hydrogel [184,185].

As the mesh size of the hydrogel approaches the size of the molecule to be released, steric hindrance increases, and the molecule of interest does not diffuse easily out of the hydrogel, due to higher friction forces [186]. Once the mesh size is smaller than the molecule size, the hindrance effect immobilizes it within the matrix, unless degradation or mesh enlargement occurs due to swelling. Although hydrogels with a very small mesh size can be engineered e.g. collagen-PEG systems [180], many GF/molecule-loaded collagen systems have a mesh size far greater than the hydrodynamic radius of the GF/molecule, and release is not influenced by swelling or degradation.

Hydrogel swelling is influenced by various external stimuli including temperature, light, glucose, salt concentrations, and pH [[186], [187], [188]]. Stimuli responding hydrogels are important when it comes to treating diseases such as cancer, as their local environment, e.g. in terms of pH, differs from healthy tissue. Collagen hydrogels that respond to pH and various salts at different concentrations have been produced in the past. Swelling increases from pH 2–4 due to the protonation of carboxyl groups into carboxylic acid groups, decreasing the repulsive forces between adjacent polymeric chains as charge is neutralized. Swelling starts decreasing again as pH increases with a substantial drop observed from pH 8–13. This is due to the formation of sodium carboxylate groups, which shield collagen fibers from absorbing water. Similarly, adding salts, especially at high concentrations has a similar shielding effect, particularly if the salts are composed of divalent or trivalent cations [188].

The diffusivity of a swollen hydrogel can be calculated using an equation by Lustig and Peppas [183]:

$$D_{s}\;=\;D_{o}\left(1-\frac{r_{s}}{\xi }\right)e^{\left[-Y\left(\frac{v_{2,s}}{1-v_{2,s}}\right)\right]}$$

D_s_ ​= ​diffusivity of swollen gel, D_o_ ​= ​diffusivity of molecule in pure solvent, r_s_ ​= ​radius of molecule, Y ​= ​ratio of the volume required for molecule movement to be possible to the average free volume per molecule of solvent (approximately 1 as the model is insensitive to the parameter), v_2,s_ ​= ​ratio between polymer volume to the swollen gel volume V_p_/V_s_, and ξ ​= ​network mesh size.

The term $\left(1-\frac{r_{s}}{\xi }\right)$ is possibly the most important and describes the probability of a molecule of radius r_s_ to diffuse through a swollen hydrogel of mesh size ξ. In fact, for a very swollen hydrogel, the equation can be simplified to:

$$D_{s}\;=\;D_{o}\left(1-\frac{r_{s}}{\xi }\right)$$

Consequently, the ratio M_t_/M_∞_ can be found as previously described. An empirical way to calculate the ratio was also developed by Peppas and Sahlin and considers both polymer relaxation and molecule diffusion during swelling, which contribute to the release profile:

$$\frac{M_{t}}{M_{\infty }}\;=\;k_{1}t^{m}+k_{2}t^{2m}$$

k_1_, k_2_, and m ​= ​geometry-dependent constants.

More complex models considering axial and radial diffusion as well as polymer dissolution have been developed but are outwith the scope of this article [185].

Another strategy used for GF release from a hydrogel is matrix degradation. As the hydrogel matrix degrades through either hydrolysis or enzymatic action (e.g. collagenase), the mesh size increases similar to the swelling scenario. Most commonly, hydrogels are permeable to water and enzymes allowing degradation to occur from both its surface and the core. If the rate of matrix bonds breaking due to degradation is higher than the rate of enzyme and water diffusion into the hydrogel, then surface degradation occurs. When the opposite is true, bulk degradation is predominant [189]. This phenomenon can be controlled by tuning the hydrophilicity of the hydrogel and its sensitivity to enzymatic degradation. While synthetic hydrogels can be easily decorated with enzyme cleavage sites and hydrophobic side chains to control these parameters, in hydrogels made from natural polymers (e.g. collagen) this fine tuning is not as straightforward and is either done through various degrees of cross-linking [50] or the introduction of a secondary non-collagenous phase [190]. The release profile from a degrading hydrogel can be calculated similarly to swelling by calculating its diffusivity at a certain timepoint. Empirical formulas for surface eroding hydrogels have also been developed by Hopfenberg [191] and Katzhendler et al. [192]. It is important to note that matrix degradation, in this case, results in an increase of the mesh size. Matrix degradation can also foster GF/molecule release when they are chemically bound to collagen, as will be discussed later.

### 3.2 Chemical cross-linking of growth factors

To avoid rapid release of GF into the extracellular space, GFs can be cross-linked to collagen. Chemical cross-linking with compounds such as N-(3-dimethylaminopropyl)-N′-ethylcarbodiimide hydrochloride (EDAC) is commonly used to cross-link collagen hydrogels but has also been used to cross-link GFs to the hydrogel to improve their biological properties. Shen et al. [193] used this technique to immobilize up to 70% of VEGF (500 ​ng/ml) on collagen compared with <10% for the non-cross-linked group. This promoted an increase in viability, invasion, and assembly of endothelial cells into the collagen hydrogel compared with the no VEGF and soluble VEGF groups. However, reactions that involve the cross-linking of GFs to substrates through primary amines can lead to reduced GF bioactivity as was observed for EGF in this study [194].

Another study involved riboflavin, a photosensitizer, and visible blue light to immobilize EGF on the surface of collagen hydrogels in a dose-dependent manner. Less than 2% of the cross-linked GF was released from the collagen substrate in PBS after 120 ​h, suggesting that a strong, covalent bond between collagen and EGF was established. Around 4% was released after 120 ​h in the presence of 0.1 and 0.2% collagenase. Collagen-EGF substrates outperformed plain collagen hydrogels in cell proliferation studies, with and without the addition of soluble EGF.

Corneal epithelial cell differentiation was also observed when EGF was immobilized on collagen substrates. Cytokeratin (CK) 3/12 – an important corneal epithelial cell differentiation marker – was upregulated and tight junctions were observed between adjacent cells. However, no significant differences were observed between the immobilized EGF and the soluble EGF group in terms of differentiation [195].

Although the primary purpose of chemically cross-linking GFs to the hydrogel matrix is to immobilize them, hydrogel degradation can lead to GF release into the extracellular space. To accurately predict the release profile, mathematical models have been developed, which account not only for mesh size change ​but also for the diffusion of matrix monomers and consequently, bound GF from the hydrogel. Models by Göpferich and Langer [196] have been developed for this purpose but are too extensive for the purpose of this article.

### 3.3 Electrostatic and other protein–protein interaction

Chemical cross-linking can be an effective method for GF presentation but can lead to protein denaturation and inactivation. A strategy that relies on the electrostatic or specific protein–protein interactions between GF and the ECM can alleviate this issue. There is substantial evidence suggesting that GF binding to the ECM regulates their activity and potency [206]**.** Bound GFs are also more resistant to proteolysis and thermal denaturation [207] and are released more gradually into the extracellular space [208]. IGFs, PDGF, VEGF, FGFs, and HGF among others have increased affinity for some ECM proteins (e.g. fibronectin) [206] as well as GAGs ​such as heparin and heparan sulfate (HS) which are large, negatively charged sulfate polysaccharides [209,210]. ECM proteins such as fibronectin have dedicated GF binding domains (e.g. FN III12-14), which also bind GFs [206], while sulphation motifs are thought to function as molecular recognition elements [210] of GAGs.

Researchers [197] showed that collagen hydrogels with HS showed a threefold increase in bFGF binding capacity and a more sustained release of the GF *in vitro* compared with collagen–hydrogels loaded with bFGF. Collagen-HS-bFGF complex showed extensive angiogenesis throughout the hydrogels over a 10-week incubation period *in vivo*, which was not the case in the collagen-bFGF and collagen–HS hydrogels [197]. Another study combined hyaluronan (HA) with collagen hydrogels, which increased their binding strength to EGF over 72 ​h, and aided in keratinocyte migration in a scratch assay together with EGF-signaling and HGF expression of fibroblasts, thus affecting keratinocyte differentiation. HA-EGF containing hydrogels also resulted in more effective wound healing [198]. Collagen hydrogels have also been functionalized with heparin giving rise to heparinized collagen matrices. These allowed for better GF (namely bFGF) binding compared with conventional collagen hydrogels [199].

Another approach for dedicated protein–protein interactions relies on modifying GFs with specialized collagen binding domains (CBDs) to increase their affinity to collagen. Egawa et al. [175] decorated GFs with CBDs similar to the ones found on other ECM proteins such as laminin and fibronectin. Plain collagen is inert for NSCs ​as it lacks sequences that present trophic or anti-apoptotic signals. Epidermal growth factor (EGF), a known mitogen for NSCs, was fused to a CBD and incorporated into cell containing collagen hydrogels. Indeed, significantly more NSCs were alive in the EGF–CBD–collagen hydrogel compared with the EGF–collagen hydrogel. Gene expression analysis also revealed that cells in the EGF–CBD–collagen expressed significantly more stem cell-, neuron-, astrocyte-, and oligodendrocyte-associated markers. Thus, coupling GFs with a CBD allowed for a more sustained release and effective presentation of the GF to cells while the plain EGF diffused out of the hydrogel too quickly to have a biological effect [175]. Although this technique alleviates the quick release problem, it requires the production of modified GFs, which can be expensive and is highly specific. In another study [200], fibronectin collagen binding domain (FNCBD) was fused to GF, namely VEGF121, to allow more effective binding to collagen type I, II, II, IV, and V substrates. FNCBD-VEGF121 showed a similar bioactivity to soluble VEGF121 but significantly increased the expression of VEGFR-2, a receptor for VEGF on endothelial progenitor cells (EPCs) confirming the importance of appropriate GF presentation to elucidate an appropriate cell response. *In vivo* experiments showed that the novel chimeric GF could induce EPC mobilization locally without having a systemic effect on the cell type.

Instead of modifying GFs, collagens with GF binding sequences from other ECM proteins can be produced recombinantly. Parmar et al. [211] developed a collagen-mimetic hydrogel system to enhance chondrogenesis. A protein containing the characteristic repeating (Gly-X-Y) backbone that can be found in collagens was expressed in *Streptococcus pyogenes*. Heparin-binding sequences were added to the collagen construct. These hydrogels displayed increased heparin absorption compared with collagen-like hydrogels without the heparin-binding sequence. The former also exhibited higher chondrogenic marker gene expression (i.e. COL2A1, ACAN, and SOX9) in MSCs cultured on the gels. This is because the heparin-binding sites present within the hydrogel bind to endogenous GFs such as TGF-β, BMPs, and bFGF, protecting them from degradation and prolonging their stability and thus biological benefit.

GF release from hydrogels that have affinity for them can also be modeled. The diffusivity can be calculated assuming that it is possible to dissociate the GF from the hydrogel using the formula [196]:

$$\frac{D}{K_{b}+1}\nabla^{2}C_{p}\;=\;\frac{\partial C_{p}}{\partial t}$$

D ​= ​diffusivity, K_b_ ​= ​concentration of bound protein/concentration of free protein ​= ​concentration of free receptor/dissociation constant, C_p_ ​= ​concentration of free protein, t ​= ​time.

This model, although useful, assumes a rapid binding mechanism and the availability of many free GF binding sites. In addition, swelling and degradation need to be considered separately.

### 3.4 Microcarrier systems

If the GF presentation and release timeline is still inadequate, the exposure of GF from a hydrogel to the extracellular environment can be further controlled by incorporating carrier systems such as GF-containing microgels into the system.

#### 3.4.1 Microgels

Microspheres or microgels have emerged in recent years as an effective type of drug delivery system, showing advantages such as tunable size, increased surface area, large attachment surface for cells, and injectability. They range from a size of a few nanometers to hundreds of microns [212,213]. A reduced size of particles makes possible their use in minimally invasive procedures. There are several methods for the production of microgels based on collagen or other biopolymers, which are reviewed in more detail in Refs. [214,215] and include emulsification [123,216], extrusion, atomization, deposition [217,218], membrane emulsification [219], microfluidics [[220], [221], [222], [223], [224]], or bioprinting [225].

Several groups have investigated collagen microgels for drug encapsulation in different tissue engineering applications. Mumcuoglu, Fahmy-Garcia et al. have described in detail a system consisting of recombinant collagen peptide microspheres encapsulating rhBMP-2 for bone regeneration [123,201,202]. The microgels, produced by emulsification and chemically cross-linked with HDMI, had a slight burst release that decreased with chemical cross-linking. All collagen compositions showed a very strong retention of the GF, with 80% of the rhBMP-2 contained after 2 weeks. Surface plasma resonance data suggest a specific interaction between the N-terminal of BMP-2 and collagen [123]. The same microsphere system encapsulating adipose-derived stromal cells was used by other groups for myocardial infarction and cardiac regeneration [216,226] ​and tested it with *in vivo* models. The authors also reported a more marked reduction of the burst release in 50 and 70 ​μm spheres compared with the 200 ​μm spheres. This behavior is not generally expected, as greater particles provide a greater diffusion pathway for encapsulated molecules, and should thus show more delayed release [227]. Nevertheless, other researchers have encountered a similar behavior in other microparticle systems ​and linked it to increased loading in particles of greater diameter. Increased initial loading capacity of bigger particles means that, once depleted of a fraction of encapsulated molecules, the particles will have an increase in porosity from the missing encapsulated molecules that compensates for their longer diffusion pathways, this way enhancing the diffusivity through their matrix [228].

Also aiming at bone regeneration, Sears et al. have reported acrylate–PEG–collagen microgels produced in a flow-focusing microfluidic device [229] ​and encapsulating an inhibitor of peroxisome proliferator-activating receptor gamma, GW9662 (GW). The same authors had previously reported pro-osteogenic MSC phenotype as a result of such inhibition by upregulation of cWnt pathway [230]. Nagai et al. encapsulated rhVEGF in collagen microspheres of 3–50 ​μm diameter, synthetized by water-in-oil emulsification and cross-linked with EDC/NHS [203]. rhVEGF was released in 8 days in 1 ​U/ml collagenase and in 4 weeks in EGM (HUVEC-specific growth medium), whereas a significantly reduced amount escaped the gels in PBS media. This was attributed to a degradation-driven mechanism of release, although the authors also point toward ​an effect of a solvent-dependent change. The authors claim GF released is bioactive on HUVEC cultures as they showed capillary formation after 21 days, comparable with a control with VEGF in solution. The same group used collagen microspheres as rhBDNF reservoirs attached to a PEG dimethacrylate membrane in a transscleral drug-delivery device for ophthalmic applications [231]. Collagen microgels have also been generated for applications other than GF delivery, like stem cell delivery [216], 3D cell culture platforms [232] or microtissue generation [220].

#### 3.4.2 Other carrier systems

A magnetic collagen scaffold was developed by Bock et al. by immersing it into an aqueous solution of ferrofluids containing iron oxide nanoparticles. These materials were designed to function as refillable, *in vivo* GF reservoirs by attracting magnetically functionalized GFs. The study showed that scaffolds supported cell adhesion and proliferation and that magnetic nanoparticles did not leak out of the scaffold over time. However, its ability to recruit GFs is still unexplored [204].

Stejskalová et al. [205] proposed a very innovative GF delivery platform, which is inspired by the large latent complex (LLC) that restrains TGF-β. During wound healing, TGF-β is deposited in an inactive state throughout the ECM, restricted by a protein complex called the LLC. As the wound heals, cells attach to the LLC via an RGD sequence releasing TGF-β, which aids in the healing process. In this study, collagen hydrogels among other scaffolds were decorated with a similar system, named Traction Force Activated Payloads (TrAP), which binds VEGF and PDGF-BB in an inactive state until it is released by a binding cell exerting force on the system. The system can be designed to selectively respond to different cell types depending on the cell binding sequence attached to the TrAP e.g. while human smooth muscle cells attach to the VAPG amino acid sequence, human dermal fibroblasts do not. However, while TrAP-PDGF-BB functionalized coverslips resulted in increased cell proliferation compared with soluble PDGF-BB in 2D, there was no significant difference between collagen hydrogels loaded with PDGF-BB and TrAP-PDGF-BB decorated collagen hydrogels in 3D. Cell differentiation, another potential application of interest, has not been explored yet.

## 4 Conclusion and future work

Hydrogel design in tissue engineering has gained a high degree of sophistication in recent years by using novel strategies for their functionalization. Collagen is one of the first biomaterials described in the tissue engineering field, and it is still chosen for its numerous advantages, together with its well-studied properties that have also gained the confidence of several regulatory bodies as an implant system.

Modification of collagen to improve its mechanical properties in terms of strength, elasticity or compliance can further expand their translation in load-bearing applications. For instance, in the production of vascular conducts already available in the clinic, Ominiflow II (LeMaitre Vascular) uses glutaraldehyde-tanned ovine collagen cross-linked with a polyester mesh that provides strength and durability to resist aneurism formation [233]. Also in the vascular engineering field, a collagen-based bioprosthesis obtained by a process of glutaraldehyde cross-linking and gamma irradiation is currently used in the clinic [234]. In terms of bone regeneration, Boston Scientific's Infuse ​uses an Achilles-tendon-derived soft collagen sponge, with hemostatic properties and whose degradability is controlled by a proprietary technology [235,236]. In the form of hydrogels, collagen is used as wound dressings to augment tissue growth and accelerate wound closure, in an application where mechanical integrity of the medical device is paramount [237]. Vergenix ​is another example of physically cross-linked collagen of recombinant source used together with platelet-rich plasma as an injectable matrix for tendinopathy and in wound healing applications [238,239].

Tissue-specific GFs are important participants in the tissue regeneration process as they dictate cell fate by participating in vital biological pathways [240]. Cells interact with the GFs in two ways: uptaking the soluble GFs from the surrounding media or interacting via receptors with GFs bound to dedicated sites (heparin domains) of some ECM proteins such as fibronectin and laminin [240]. Collagen, a widely used substrate for tissue engineering, does not exhibit these dedicated binding sites but has still been commercialized for use in conjunction with GFs [175], e.g. Infuse ​from Medtronic BMP-2 [241] and Regranex ​from Smith&Nephew PDGF [242]. These products have been successful in regenerating bone and treating ulcers, respectively, but the amount of GF loaded (1.5 ​mg/ml BMP2 [241] and 100 ​μg/g PDGF [242], respectively) far surpasses physiological levels of the GFs, resulting in side-effects such as ectopic bone formation and malignant growths [243,244]. Consequently, there is a real clinical need to develop new methods, in collagen-based materials, to present GFs locally at low concentrations, which have the same biological effect as the current state-of-the art. Particularly promising approaches rely on custom GFs covalently cross-linked with CBD sequences [175,200]. Incorporating different molecules such as GAGs into the collagen matrix is another simple but effective approach ​because GAGs such as hyaluronan and chondroitin have higher affinity to some GFs compared with collagen [197,198]. More advanced carrier systems, such as TrAP, have also been engineered to deliver GFs in-situ using peptides triggered by forces exerted by cells to their local microenvironment [205].

The ability to control the mechanical properties and degradation of collagen hydrogels and sustain GF release ​is a great tool to optimize systems for use in different tissue environments. This is confirmed by *in vitro* and *in vivo* studies, which demonstrate that newly developed collagen systems have the potential to be used in clinical studies in the future. Still, studies using collagen systems, with exceptions (e.g. Ref. [78]), often approach the topic from a one-sided perspective and do not consider both mechanical and biological stimuli (e.g. GF loading) simultaneously.

Collagen hydrogels in tissue engineering have seen massive improvements in recent years that have laid important groundwork for future developments. However, these hydrogels still lack essential properties of the native ECM. For instance, similarly to other systems in the field, encapsulated co-cultures within the gel or the utilization of combinations of GFs at optimized concentrations for tissue growth are still in its infancy. Furthermore, GF release from collagen gels typically include an initial burst release. It is urgent to engineer collagen hydrogels that offer full control on the release kinetics of GFs, to recapitulate sustained local concentrations found *in vivo*. Finally, the scalability, shelf life, and handling of collagen hydrogels needs to be analyzed and improved for them to become the material of choice for surgeons in the field.

## Declaration of competing interest

The authors declare that they have no known competing financial interests or personal relationships that could have appeared to influence the work reported in this paper.
